# Low-thermal-budget electrically active thick polysilicon for CMOS-First MEMS-last integration

**DOI:** 10.1038/s41378-024-00678-5

**Published:** 2024-06-06

**Authors:** Aron Michael, Ian Yao-Hsiang Chuang, Chee Yee Kwok, Kazuo Omaki

**Affiliations:** UNSW, Sydney, NSW 2052 Australia

**Keywords:** Electrical and electronic engineering, Materials science, Other nanotechnology

## Abstract

Low-thermal-budget, electrically active, and thick polysilicon films are necessary for building a microelectromechanical system (MEMS) on top of a complementary metal oxide semiconductor (CMOS). However, the formation of these polysilicon films is a challenge in this field. Herein, for the first time, the development of in situ phosphorus-doped silicon films deposited under ultrahigh-vacuum conditions (~10^−9^ Torr) using electron-beam evaporation (UHVEE) is reported. This process results in electrically active, fully crystallized, low-stress, smooth, and thick polysilicon films with low thermal budgets. The crystallographic, mechanical, and electrical properties of phosphorus-doped UHVEE polysilicon films are studied. These films are compared with intrinsic and boron-doped UHVEE silicon films. Raman spectroscopy, X-ray diffraction (XRD), transmission electron microscopy (TEM) and atomic force microscopy (AFM) are used for crystallographic and surface morphological investigations. Wafer curvature, cantilever deflection profile and resonance frequency measurements are employed to study the mechanical properties of the specimens. Moreover, resistivity measurements are conducted to investigate the electrical properties of the films. Highly vertical, high-aspect-ratio micromachining of UHVEE polysilicon has been developed. A comb-drive structure is designed, simulated, fabricated, and characterized as an actuator and inertial sensor comprising 20-μm-thick in situ phosphorus-doped UHVEE films at a temperature less than 500 °C. The results demonstrate for the first time that UHVEE polysilicon uniquely allows the realization of mechanically and electrically functional MEMS devices with low thermal budgets.

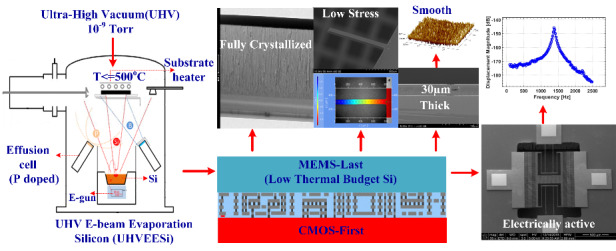

## Introduction

Due to its excellent mechanical and electrical properties, silicon is clearly the dominant material used for forming mechanical and electrical devices in microelectromechanical systems (MEMS). Silicon does not exhibit mechanical hysteresis. The electrical properties of this material can be controlled easily and precisely by selectively doping n- and p-type impurities. Furthermore, silicon is a relatively inexpensive material that is amenable to batch manufacturing^1^. This material is central to the field of integrated circuit technology, particularly in the development of CMOS making CMOS–MEMS monolithic integration necessary for the creation of relatively small and highly functional systems. Various kinds of CMOS–MEMS monolithic integration approaches have been proposed and investigated. Depending on the stage at which the MEMS process is integrated into the CMOS process, the existing monolithic integrations are classified as MEMS-first, MEMS-intermediate, or MEMS-last approaches^2^. The MEMS-last approach has the advantage of modular integration. In this process, the CMOS and MEMS can be independently developed in separate foundries with the MEMS built on top of the CMOS. Therefore, the key requirement in MEMS-last integration is minimization of the thermal budget required to realize the MEMS component. Minimizing the thermal budget can prevent CMOS circuitry damage and maintain system integrity. According to experimentation, the main impacts of post-CMOS thermal exposure are not on the transistor performance but on the increase in sheet resistance of the metal alloy and the interconnection stack used in CMOS technology. A 0.35-μm CMOS can withstand a temperature of 525 °C for 90 min^3^. A 0.25-µm CMOS can withstand thermal processing at 425 °C for 6 h, 450 °C for 1 h and 475 °C for 0.5 h^4^. A thermal budget of 2 h at 500 °C is established for BEOL integration on top of a 28-nm CMOS^5^. The requirement of such a stringent low-thermal-budget process significantly restricts the MEMS-last approach, as silicon requires high-temperature processing to exhibit low stress, full crystallization, and electrical activity. Existing polysilicon deposition techniques require temperatures greater than 600 °C to achieve full crystallization and electrical activation of dopants^6^. Metal films can be deposited at low temperatures by sputtering^7^ and electroplating^8,9^. However, these films exhibit mechanical hysteresis and pose a challenge when narrow high-aspect-ratio structures are formed. Other promising low thermal budget materials include low-pressure chemical-vapor-deposition-based silicon germanium (LPCVD poly-SiGe)^10^ and plasma-enhanced chemical-vapor-deposition-based (PECVD) silicon germanium (SiGe)^11^, which are formed with low thermal budgets due to the introduction of Ge. Ge lowers the activation energy for crystallization. A gyroscope formed from PECVD SiGe has been demonstrated for MEMS-last integration^11^. However, PECVD SiGe films are susceptible to humidity and atmospheric corrosion, leading to long-term reliability issues and surface roughness. LPCVD poly-SiGe materials are reported to have relatively high compressive residual stresses^12^ and to be susceptible to buckling. Furthermore, these materials are not conducive to thick film formation due to the requirement of high-thermal-budget. Electron-beam evaporated polysilicon films can be deposited at high deposition rates and low substrate temperatures. However, scholars have previously focused mainly on solar cell applications^13,14^, where silicon films are deposited on contact layers, such as ZnO:Al. As a result, the mechanical characteristics, such as residual stress, stress gradient, surface morphology and Young’s modulus, which are crucial to MEMS applications, have not yet been thoroughly investigated. Reportedly, e-beam-evaporated intrinsic silicon films deposited under ultrahigh-vacuum conditions demonstrate signs of early crystallization^15^. However, since the silicon films are intrinsic, they can be used only as structural layers and have no electrical characteristics. In this work, in situ phosphorus doping from an effusion cell is introduced to form an electrically active polysilicon film at various substrate deposition temperatures. The crystallographic, mechanical, and electrical properties of phosphorus-doped silicon films that are relevant to MEMS are investigated. The films are compared with intrinsic, boron-doped silicon films. The results show that mechanically and electrically active and fully crystalline silicon can be formed at low temperatures (under 500 °C) under ultrahigh-vacuum conditions using e-beam-evaporated phosphorus-doped silicon. Then, a comb-drive structure is fabricated using the polysilicon film, and its performance is characterized as an actuator and accelerometer, demonstrating its potential as a silicon material solution for post-CMOS MEMS integration.

## Results

### Crystallization

The percentage of crystallization of the UHVEE silicon films is determined from Raman spectroscopy measurements. The Raman spectra of the silicon films can be separated into 3 major parts: (i) a broadband band centered at approximately 480 cm^−1^, which represents amorphous and disordered structures; (ii) a narrow band centered at 518 cm^−1^, which represents crystallized silicon; and (iii) a weak peak at 500 cm^−1^, which represents the presence of hexagonal silicon^16^. A Gaussian peak is fitted onto each of the three bands, with *A* denoting the area enveloped by the Gaussian curve. The percentage of crystallization is subsequently calculated based on the following equation:

$${A}_{{C}_{{RT}}}=\frac{{A}_{520}+{A}_{500}}{{A}_{520}+{A}_{500}+0.8{A}_{480}}{\rm{x}}100 \%$$

**Fig. 1:**
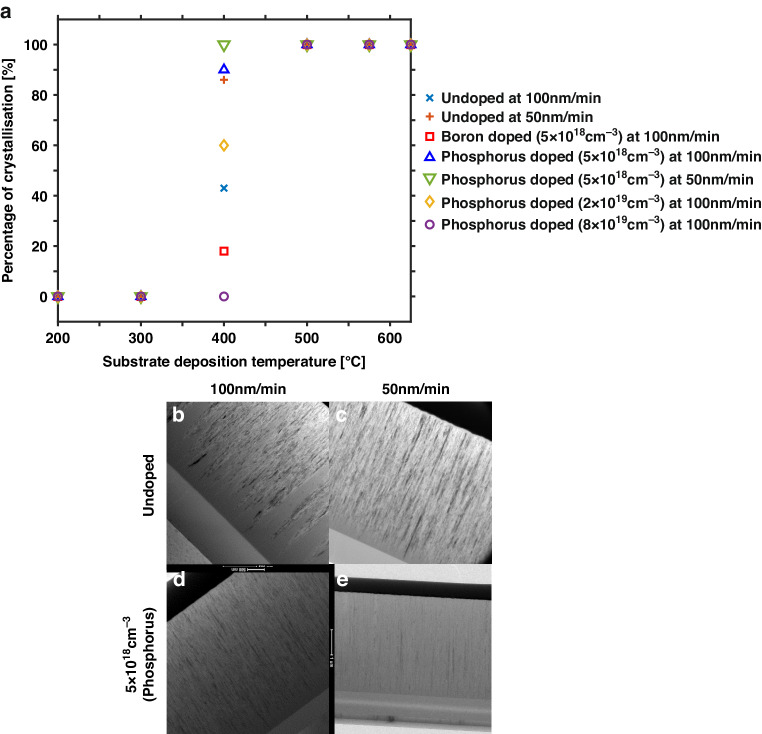
Influence of doping, deposition temperature and rate on the crystallization level and microstructure profile of UHVEE films. **a** Percentage of crystallizations for undoped, boron-doped (5 × 10^18^/cm^3^) and phosphorus-doped (5 × 10^18^/cm^3^, 2 × 10^19^/cm^3^, 8 × 10^19^/cm^3^) specimens at 50 nm/min and 100 nm/min deposition rates. Note that the data points for the various silicon films processed at/above 500 °C and below 400 °C overlap; X-TEM microstructures of the UHVEE silicon films deposited at 400 °C: **b** undoped specimen at 100 nm/min; **c** undoped specimen at 50 nm/min; **d** phosphorus-doped specimen (5 × 10^18^/cm^3^) at 100 nm/min; and **e** phosphorus-doped specimen (5 × 10^18^/cm^3^) at 50 nm/min

The 0.8 factor from the 480 cm^−1^ peak is used to consider the relatively high level of phonon excitation from amorphous silicon^13^. Then, the percentage of crystallization of the as-deposited silicon films is plotted with respect to various substrate deposition temperatures and doping levels, as shown in Fig. 1a.

Silicon films evaporated at substrate deposition temperatures below 300 °C are entirely amorphous. When the substrate deposition temperature is increased to a value above 500 °C, the films crystallize completely. For substrate deposition temperatures between 300 °C and 500 °C, the silicon films partially crystallize. At a substrate deposition temperature of 400 °C, the undoped film exhibits only 43% crystallization. Doping the silicon film with phosphorus or boron modifies the percentage of crystallization. The introduction of boron dopants tends to retard early crystallization. The percentage of crystallization is reduced from 43% for undoped films to less than 20% for 5 × 10^18^/cm^3^ boron-doped films. On the other hand, introducing a modest amount of phosphorus dopants enhances the level of crystallization. A silicon film with a phosphorus doping level of 5 × 10^18^/cm^3^ displays 90% crystallization compared to 43% crystallization for the film without doping. The increase in the percentage of crystallization with the introduction of phosphorus dopants into UHVEE silicon may be explained by the increasing effect of phosphorus dopants on the surface diffusivity of silicon atoms^17^. This phenomenon is attributed to the relatively high kinetic energy imparted by the evaporated phosphorus atoms from the effusion cell to the silicon atoms on the surface of the substrate. Thus, the silicon atoms are more likely to diffuse and be incorporated into the silicon crystal grains as more phosphorus atoms are introduced with increasing doping. A further increase in the phosphorus doping concentration, however, has the opposite effect of retarding crystallization. When the phosphorus doping level is increased from 5 × 10^18^/cm^3^ to 2 × 10^19^/cm^3^, the percentage of crystallization is reduced from 90% to 60%, as shown in Fig. 1a. When the phosphorus doping concentration is increased to 8 × 10^19^/cm^3^, the percentage of crystallization further decreases to zero, and the film becomes completely amorphous at 400 °C. An increase in the phosphorus doping level may lead to an alloying effect^18,19^, which increases the activation energy required to form silicon crystals and hence retards the crystallization process.

Crystallization is also affected by the evaporation rate. The percentage of crystallization at a phosphorus doping level of 5 × 10^18^/cm^3^ increases from 90% to 100% as the evaporation rate decreases from 100 nm/min to 50 nm/min. The effect of the phosphorus doping level and evaporation rate on the crystallization of the UHVEE silicon films can clearly be observed from the X-TEM images presented in Fig. 1b–e. Figure 1b–e show the effects of the phosphorus doping level and evaporation rate on the Si/SiO_2_ interface. Figure 1b shows that there is an apparent amorphous silicon region at the Si/SiO_2_ interface for the undoped silicon film evaporated at 100 nm/min. When the evaporation rate is reduced to 50 nm/min, the amorphous silicon region at the interface decreases, as shown in Fig. 1c. When the silicon film is evaporated at 100 nm/min and in situ phosphorous doped, the amorphous silicon region at the interface is significantly reduced, as shown in Fig. 1d. The amorphous silicon region at the interface disappears completely in Fig. 1e when the evaporation rate is reduced to 50 nm/min, and the film is doped with phosphorus in situ. The results show that fully crystallized polysilicon films can be obtained with low thermal budgets at 400 °C, which is suitable for CMOS-first and MEMS-last integration because of the ability to control the in situ phosphorus doping level and evaporation rate. The increase in crystallinity achieved by reducing the deposition rate may be explained by the promotion of highly ordered film formation and nucleation rates^18^ as the evaporated silicon atoms have sufficient time on the surface to move and contribute to grain growth.

**Fig. 2:**
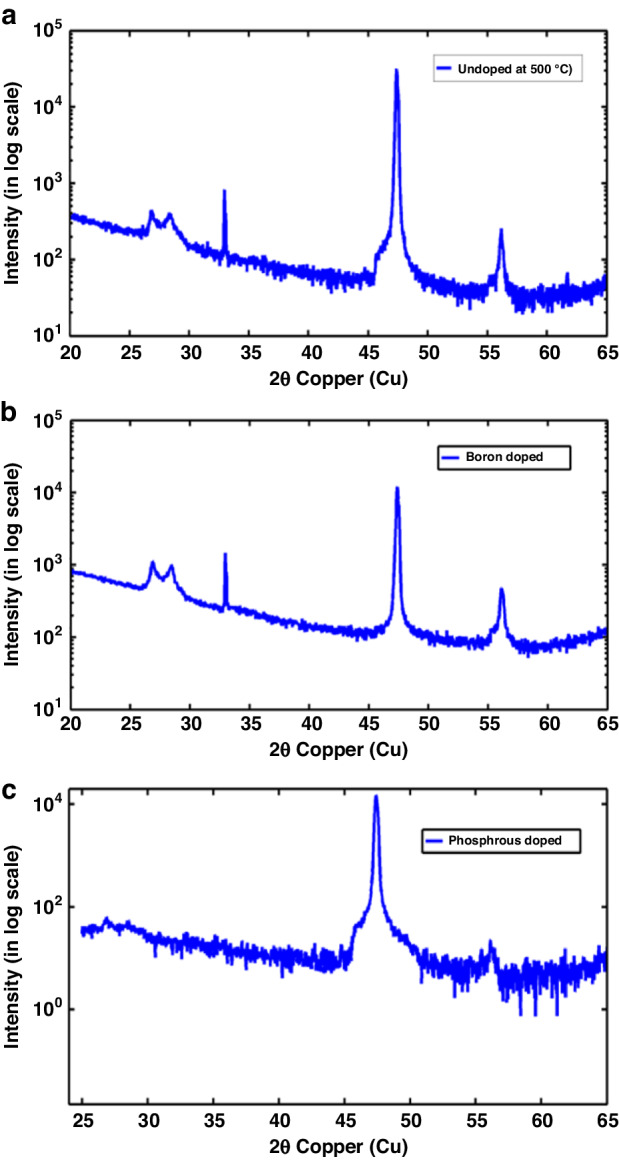
Effect of doping type on the grain orientations of the UHVEE films. XRD patterns of **a** undoped, **b** boron-doped, and **c** phosphorus-doped UHVEE polysilicon films

X-ray diffraction (XRD) is performed on the UHVEE polysilicon films to determine the crystallographic orientation of the silicon grains. Figure 2a–c show the XRD patterns of undoped, boron-doped, and phosphorus-doped UHVEE silicon films deposited at 500 °C. The XRD patterns indicate that the UHVEE silicon films have peaks at 28.5°, 47.4° and 56.0° that correspond to grains with crystal orientations of (111), (110) and (311), respectively, with the (110) orientation being the most dominant. This XRD behavior is very similar to that of other e-beam-evaporated silicon films reported for solar cell applications^7^. The additional peak close to the (111) peak at 26.9° is attributed to an expansion of the (111) lattice plane at the grain boundaries^20^. The peak can be observed in films that undergo a transition from a-Si to c-Si. By comparing the XRD characteristics of the boron-doped film with those of the undoped film, it is found that the boron-doped films (Fig. 2b) have a slightly lower (110) peak, implying slight delay of crystal growth in the (110) orientation. Conversely, the phosphorus-doped UHVEE polysilicon films in Fig. 2c have (110)-oriented grains with no (111)-oriented grains and a peak at 26.9°, implying that phosphorus promotes crystallization. This implication is supported by the Raman spectroscopy results showing that the percentage of crystallization is enhanced by in situ phosphorus doping.

**Fig. 3:**
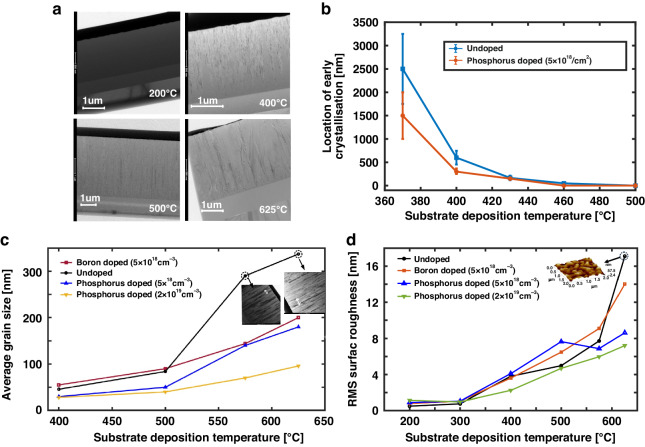
Study of the grain structure and surface morphology behaviour of the UHVEE films. Microstructure of UHVEE polysilicon: **a** X-TEM image of the film; **b** location of early crystallization; **c** average silicon grain size; and **d** RMS surface roughness

Cross-sectional transmission electron microscopy (X-TEM) is used to further investigate the crystal texture of the film. A platinum layer is deposited onto the UHVEE polysilicon film to protect its surface, while focused ion beam (FIB) is used to prepare a sample for X-TEM. The platinum layer appears as a top dark layer covering the silicon in the X-TEM images. In Fig. 3, the X-TEM images show in situ phosphorus-doped (5 × 10^18^/cm^3^) UHVEE silicon films deposited at 200 °C, 400 °C, 500 °C, and 625 °C. Figure 3a shows that the undoped silicon deposited at 200 °C is entirely amorphous and partially amorphous at 400 °C, while the undoped silicon films are fully crystallized at a substrate deposition temperature of 500 °C and above. The X-TEM images clearly show that the crystal grains have columnar structures, resulting from the highly distinctive (110)-oriented texture^21^.

The average distances above the Si/SiO_2_ interfaces at which crystallization is initiated in the phosphorus-doped UHVEE silicon films at various substrate temperatures are measured from the X-TEM images and plotted, as shown in in Fig. 3b. The distances represent the thickness of the amorphous layer above the interface. As shown in the plot, as the deposition temperature increases, the thickness of the amorphous layer at and above the Si/SiO_2_ interface decreases. At deposition temperatures below 400 °C, a very sharp decrease in the amorphous layer thickness can be observed. The amorphous layer decreases from 1500 nm to 200 nm with a small increase in temperature from 370 °C to 400 °C. When the substrate temperature is increased to 460 °C, the amorphous layer completely disappears. These results from the X-TEM images correlate well with the results of the percentage of crystallization obtained from Raman spectroscopy.

According to the X-TEM images in Fig. 3a, the columnar grain sizes are significantly influenced by the substrate deposition temperature. When a sample is deposited at a high temperature, relatively coarse and large grains are yielded. To compare the grain sizes, the average widths of the columnar grains are measured via X-TEM and are plotted in Fig. 3c. From the figure, the silicon films deposited at relatively low temperatures reaching 500 °C have fine columnar structures with small crystal sizes. For these films, the early crystallization mechanism is driven by the kinetic energy of the e-beam-evaporated silicon atoms^15^. In addition, the thermal energy from substrate heating contributes to the growth of silicon films. This contribution can be seen from the gradual increase in grain size as the temperature increases. However, for temperatures above 575 °C, the X-TEM images show that the crystal grains become increasingly coarse and large, suggesting a change in the grain formation mechanism. The coarse silicon grain structure suggests that the thermal energy from substrate heating is the dominant mechanism of crystallization, as the kinetic energy of the e-beam-evaporated silicon remains the same. Reportedly, LPCVD silicon begins to crystallize from thermal energy at a temperature of 570 °C^21,22^. This trend is in good agreement with the temperature growth rate of the crystal grains of UHVEE polysilicon. Interestingly, phosphorus-doped UHVEE polysilicon films exhibit finer crystal grains than undoped and boron-doped films. The average grain size is reduced by half from 340 nm to 170 nm at 625 °C when the UHVEE polysilicon is doped with 5 × 10^18^/cm^3^ phosphorus in situ. A further decrease in grain size is observed when the phosphorus doping level is increased. The average grain size is reduced by almost half, from 170 nm to 90 nm, after the film undergoes in situ phosphorus doping of 2 × 10^19^/cm^3^. This property of in situ phosphorus doping is beneficial when polysilicon films with relatively smooth surface morphologies are needed. However, these fine-grain films may not be ideal for some applications, such as for fabricating high-mechanical-quality resonators, because the high grain boundary density in these films leads to great internal material loss^23,24^, which compromises the mechanical quality. For these applications, coarse grains that can be achieved at high deposition temperatures or with boron doping are preferred to mitigate the effects of grain boundaries. Despite the internal material loss in fine-grain in situ phosphorus-doped films, these films can be appealing for use in resonator applications relative to single-crystal silicon and other polysilicon technologies. These films can enable CMOS-first MEMS-last-integration, thick polysilicon formation and wafer-scale vacuum packaging, leading to reductions in power consumption, cost, and size.

### Surface morphology

The surface morphologies of undoped, phosphorus-doped and boron-doped UHVEE silicon films are measured and plotted, as shown in Fig. 3d. The amorphous silicon films deposited under 300 °C exhibit very smooth surface morphologies with RMS roughness values of less than 1 nm. As the UHVEE silicon films are deposited between 300 °C and 500 °C, the silicon films become partially crystalline with a mixture of amorphous and grain textures, leading to a substantial increase in surface roughness. Compared with those of phosphorus-doped silicon films, undoped and boron-doped silicon films display smooth surface textures in the temperature range of 300 °C–500 °C. This difference may be explained by the relatively low percentage of crystallization exhibited by the undoped and boron-doped silicon films, as observed from Raman spectroscopy. After the first jump, the surface roughness increases linearly with the deposition temperature until it reaches 575 °C, when a second jump is observed for the boron-doped and undoped UHVEE silicon films. The second jump correlates well with the formation of coarse silicon films due to the change in the crystallization mechanism from kinetic to thermal energy. Interestingly, the second jump is not observed in the phosphorus-doped silicon films, which display gradual linear increases in surface roughness, yielding smoother surface morphologies at 625 °C than the boron-doped and undoped silicon films. The surface roughness of the silicon film doped with phosphorus at 5 × 10^18^/cm^3^ and deposited at 625 °C is less than 8 nm. The surface roughness is reduced to 6 nm when the phosphorus doping level is increased to 2 × 10^19^/cm^3^.

### Mechanical properties

**Fig. 4:**
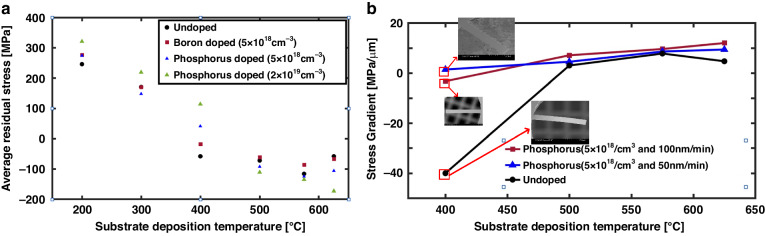
Impact of doping and deposition temperature on the stress characteristics of the UHVEE films. **a** Average residual stress and **b** stress gradient at various substrate deposition temperatures

The average residual stresses of the films are determined from the wafer curvature measurements. The residual stresses of the phosphorus-doped polysilicon films with respect to the deposition temperature are plotted in Fig. 4a, along with those of the undoped and boron-doped silicon films for comparison. The amorphous phosphorus-doped silicon films exhibit higher tensile stresses than the undoped and boron-doped silicon films. As the films crystallize with increasing deposition temperature, the films become less tensile and then more compressive.

At deposition temperatures of at least 500 °C, the compressive stresses in the films can be further enhanced by phosphorus doping. The compressive stress at a deposition temperature of 500 °C increases from −72 MPa for the undoped film to −92 MPa for the in situ phosphorus-doped film with a concentration of 5 × 10^18^/cm^3^ and to −111 MPa with a concentration of 2 × 10^19^/cm^3^. Heavily phosphorus-doped polysilicon film is known to exhibit a greater magnitude of compressive residual stress due to the accumulation of excessive phosphorus at grain boundaries^25^. Although these compressive residual stress levels are notably greater than those of the undoped films, they are still considered low. Interestingly, the average residual stress of the phosphorus-doped (5 × 10^18^/cm^3^) silicon film deposited at 400 °C is 41 MPa, which is a small, slightly tensile, and desirable characteristic for many mechanical structures.

In addition to the low average residual stress, a low residual stress gradient is another critical mechanical property that silicon films should exhibit when forming mechanical structures. To measure the residual stress gradient of phosphorus-doped (5 × 10^18^/cm^3^) UHVEE polysilicon films, 4-μm-thick cantilever beams ranging from 300 μm to 900 μm in length are released via deep reactive ion etching (DRIE) from both sides of the substrate. This process is followed by HF wet etching to remove the thermal oxide on which the UHVEE polysilicon films is deposited. A PolyTech MSA500 is used to determine the deflection profile and resonance frequency, from which the stress gradient and Young’s modulus values of the silicon films are obtained. No significant change in Young’s modulus can be observed for the doped or undoped silicon films. The Young’s modulus is approximately 160 GPa, which is close to that of (110)-oriented single-crystal silicon. From the out-of-plane deflection profile of the released cantilever beams, the radius of curvature, R, is obtained from the equation and the stress gradient,*S*_*σ*_ and is subsequently calculated by^26^ ${S}_{\sigma }=\frac{1}{2}\frac{{EH}}{R}$, where E is the Young’s modulus and H is the film thickness. The extracted stress gradients of the films are plotted, as shown in Fig. 4b. If a positive stress gradient exists in the cantilever, the cantilever beam bends upward, implying that the bottom layer of the film is more compressive than the top layer. If a negative stress gradient exists in the cantilever structure, the beam bends downward as the top layer becomes more compressive than the bottom. For phosphorus-doped silicon films, the stress gradient is reduced significantly at 400 °C from −39.8 MPa/μm (undoped) to −3 MPa/μm (5 × 10^18^/cm^3^ phosphorus doping). A further reduction in the stress gradient to 1.2 MPa/μm is obtained when the deposition rate is reduced to 50 nm/min. The source of the stress gradient is the variation in the crystallization state across the thickness of the evaporated silicon films, which can be observed from the X-TEM images of the silicon films (in Figs. 1b–e and 3a). The partially crystalline silicon films have cross-sectional profiles with amorphous states near their Si/SiO_2_ interfaces and fully crystalline states near their surfaces. The bottom section in the amorphous state experiences tensile stress, while the top section in the fully crystalline state exhibits compressive stress. These characteristics result in negative stress gradients in the silicon films, as shown in Fig. 4b, at 400 °C. As the substrate deposition temperature increases and the films become fully crystalline, the bottom section is no longer in an amorphous state and becomes crystalline with compressive stress, reducing the stress gradient significantly.

### Electrical properties

Electrically active polysilicon films are needed to form functional MEMS devices and components. Undoped UHVEE silicon, with its large resistivity, is not electrically functional. To give electrical functionality to UHVEE polysilicon, in situ boron and phosphorus doping is introduced into the film deposition process. UHVEE polysilicon films with boron doping levels of 5 × 10^18^/cm^3^ and phosphorus doping levels of 5 × 10^18^/cm^3^ and 2 × 10^18^/cm^3^ are deposited at various substrate temperatures, and their resistivities are measured. The measured resistivities are provided in Table 1. From the measurements, amorphous films deposited at temperatures below 400 °C have very large resistivities. As the substrate deposition temperature increases, the resistivity of the silicon films decreases significantly when the films become more electrically active. The substrate deposition temperature at which electrical activation occurs depends on the doping type and level. For both the boron-doped (5 × 10^18^/cm^3^) and phosphorus-doped (2 × 10^19^/cm^3^) samples, electrical activation occurs at 500 °C. However, early activation at 400 °C is observed for of the 5 × 10^18^/cm^3^ phosphorus-doped samples. This finding aligns well with the observed fully crystalline nature of phosphorus-doped UHVEE silicon films evaporated at 50 nm/min.

**Table 1:** Resistivities of UHVEE polysilicon films (50 nm/min) [Ω-cm]

Doping	200 °C	300 °C	400 °C	450 °C	500 °C	575 °C	625 °C
**Boron (5 x 10**^**18**^ **cm**^**−3**^**)**	668	436	1340	1200	2.1	0.2	0.1
**Phosphorus (5 x 10**^**18**^ **cm**^**−3**^**)**	510	380	17.5	1.75	1.25	1.12	0.82
**Phosphorus (2** **×** **10**^**19**^ **cm**^**−3**^**)**	116	90	140	100	0.445	0.08	0.03

### Comb-Drive Structure

**Fig. 5:**
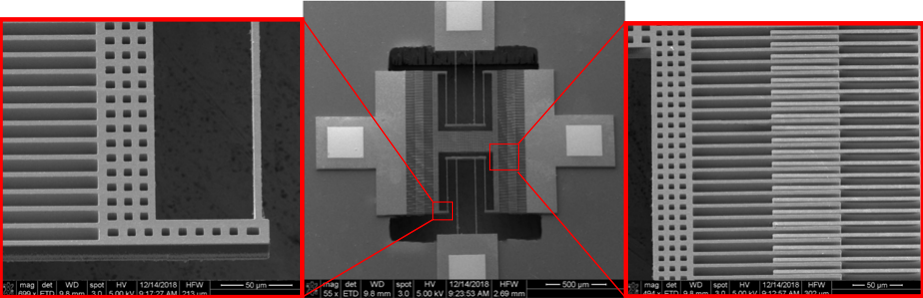
SEM images of the fabricated Comb-Drive

SEM images of the successfully fabricated comb-drive structures as an actuator and accelerometer using 20-µm-thick phosphorus-doped (5 × 10^18^/cm^3^) UHVEE polysilicon films deposited at 450 °C are shown in Fig. 5. According to the SEM images, the released comb-drive fingers are flat, displaying no apparent out-of-plane bending. This characteristic indicates that the UHVEE polysilicon film has a low stress gradient. The stress gradient bends the released structures, leading to out-of-plane deflection^27^. The existence of a stress gradient can cause the overlapping fingers of the comb-drive to have different out-of-plane deflections. This phenomenon is clearly not the case, as the interdigitated fingers remain relatively flat with insignificant out-of-plane displacement offsets between them.

**Fig. 6:**
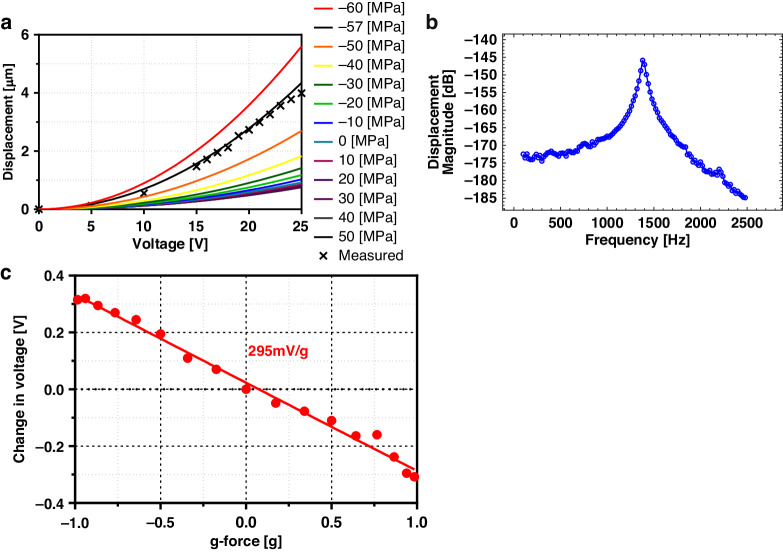
Characterisation of the comb-drive structure fabricated from thick UHVEE polysilicon as accelerometer and resonator. Characterization of the comb-drive structure: **a** static; **b** dynamic; and **c** readout circuit output for sensed accelerations

To measure the static characteristics of the comb-drive structure, the rotor of the comb-drive is grounded while applying a DC voltage to one side of the comb-drive stator. The DC voltage is increased while the generated in-plane displacements of the rotor are measured using a Polytec-MSA500. The measured in-plane displacements are plotted along with the simulated results from finite element analysis for various stress levels in the film, and the results are presented in Fig. 6a. The measured results are in good agreement with the simulated results when a small compressive stress of 57 MPa is considered in the film. This result agrees well with the residual compressive stress expected from in situ phosphorus-doped UHVEE polysilicon residual stress measurements. The results also show that the in-plane displacement of the comb-drive is a parabolic function of the applied voltage, as expected from theory, indicating that the comb-drive fabricated from in situ phosphorus-doped UHVEE polysilicon at a low thermal budget is electrically and mechanically functional like those fabricated from single-crystal or high-temperature silicon.

A strobe-light analyzer module on the Polytec-MSA500 is used to investigate the dynamic performance of the comb-drive. A static DC voltage of 5 V is applied to the stator of the comb-drive to bias the comb structure, while an AC signal of 0.5sin (*ωt*)V is applied to the rotor of the comb-drive for excitation.

The frequency of the AC signal is swept from 100 Hz to 2500 Hz in 20 Hz increments. While the comb-drive is driven by AC signals at different frequencies, the strobe light analyzer records the resulting in-plane displacements of the comb-drive. The measured in-plane frequency response of the comb-drive is plotted in Fig. 6b. The resonant frequency of the fabricated comb-drive structure occurs at 1380 Hz. When the comb-drive structure is simulated without stress, the resonance frequency is 2120 Hz. The large discrepancy between the measured and simulated results is attributed to the existing small compressive residual stress in the UHVEE polysilicon. The resonant frequencies of the comb-drive at various residual stresses are obtained from COMSOL simulations. An increase in the residual compressive stress causes the comb-drive to become less stiff, hence leading to a reduction in the resonance frequency. When a 60-MPa compressive stress is applied to the polysilicon film, the resonance frequency is simulated to be 1550 Hz, which is close to the measured resonance frequency of 1380 Hz. The small discrepancy between the measurement and simulation results may arise due to fabrication imperfections, such as slight lateral under etch and footing effects^28^, which are not considered in the simulation.

The comb-drive structure is used to measure acceleration to demonstrate its application as an accelerometer. The device is connected to a read-out circuit and exposed to acceleration in the range of g to -g. The structure has a linear sensitivity of 290 mV/g, as shown in Fig. 6c, demonstrating the promising functionality of UHVEE polysilicon as a material for fabricating an inertial sensor with a low thermal budget. Unlike previous comb-drive structures made from single-crystal silicon and polysilicon films deposited with high thermal budgets, this is the first comb-drive structure composed of thick polysilicon with a low thermal budget.

## Discussion

Materials suitable for monolithic CMOS-first integration should exhibit excellent electrical and mechanical properties at low thermal budgets when deposited as films and further processed. These materials should enable the formation of thick films with smooth surface morphologies. Only a limited number of materials display these characteristics. Metal films exhibit mechanical hysteresis and pose a challenge when narrow high-aspect-ratio structures are formed. Plasma-enhanced chemical-vapor-deposition-based (PECVD) silicon germanium (SiGe) films are susceptible to humidity and atmospheric corrosion, leading to long-term reliability issues and rough surfaces. These issues are overcome by the phosphorus-doped UHVEE polysilicon films reported in this work. The phosphorus-doped and thick UHVEE silicon films are fully crystalline electrically active polysilicon films with low average residual stresses, low stress gradients and smooth morphologies that can be achieved at deposition temperatures less than 500 °C. This temperature is significantly lower than that required for silicon films formed by other deposition techniques, such as sputtering, PECVD and LPCVD; these films are known to fully crystallize at temperatures above 600 °C^6,17,18,29,30^. The introduction of phosphorus dopants into UHVEE silicon is responsible for the observed enhancement of early crystallization, which may be explained by the increase in the surface diffusivity of silicon atoms with phosphorus doping^19^. Unlike undoped and boron-doped UHVEE silicon and other silicon films, such as LPCVD- or PECVD-based films, the phosphorus-doped UHVEE silicon films do not show (111)-oriented grains or an associated peak close to (111). The grains are predominantly in the (110) orientation. This high monotonicity in grain orientation may be responsible for the promotion of crystallization with the introduction of phosphorus dopant. The crystallization is further enhanced by reducing the evaporation rate, which provides evaporated silicon atoms sufficient time to diffuse and participate in grain growth, promoting highly ordered film formation and nucleation^31^. However, care should be taken when phosphorus doping is used to avoid causing an alloying effect that tends to increase the activation energy required for silicon crystal formation^32,33^, retarding early crystallization. Early crystallization in phosphorus-doped UHVEE films leads to the development of compressive stress, which may be attributed to competitive grain growth^25,34^. The average residual compressive stress is relatively low at less than 120 MPa. This low compressive stress is a useful attribute of the UHVEE deposition technique and enables the deposition of thick polysilicon films. The realization of a comb-drive structure made of a 20-µm-thick polysilicon film in this work demonstrates the usefulness and potential of this technology. We have demonstrated the feasibility of using 30-µm- and 60-µm-thick phosphorus-doped polysilicon films. Depositing thick polysilicon films at less than 500 °C using other silicon deposition techniques, such as PECVD, LPCVD or sputtering, is extremely challenging because they are prone to peeling due to the development of a high tensile stress and a low deposition rate; thus, these materials are not suitable for thick film deposition. In comparison, UHVEE silicon can be evaporated at a deposition rate approaching 1 µm/min. The compressive stress generally decreases with increasing deposition rate. The combination of a high deposition rate and low compressive stress is key for enabling significantly thick UHVEE polysilicon layers with low thermal budgets. Another unique attribute of early crystallization in phosphorus-doped films is the formation of relatively fine grains, resulting in a significantly smoother polysilicon surface morphology than that of polysilicon films deposited by other methods, including LPCVD. Fully crystallized LPCVD polysilicon films deposited at 610 °C have RMS roughness of 71 nm^35^. In comparison, UHVEE silicon films provide a significantly smoother surface morphology of less than 7 nm at the corresponding deposition temperature. The results highlight the advantages of UHVEE silicon films, including their ability to yield smooth films without polishing.

Early crystallization results in phosphorus-doped UHVEE films with significantly reduced residual stress gradients. By investigating the Si/SiO_2_ interfaces of partially crystallized UHVEE silicon films (Fig. 1b–e and Fig. 3c), we can observe crystal grains on top of the amorphous layer at and above the Si/SiO_2_ interface, indicating that crystal nucleation is initiated above the Si/SiO_2_ interface. This phenomenon is in contrast with the crystallization behavior of partially crystallized LPCVD polysilicon, where nucleation is initiated at the Si/SiO_2_ interface^35,36^ and an amorphous layer on top of crystal grains is observed. This difference may imply that the crystallization mechanisms of UHVEE polysilicon and LPCVD silicon are different. The crystallization profile of the UHVEE polysilicon film may be attributed to several factors. (i) The crystal formation of the polysilicon film is driven by the kinetic energy provided by evaporated e-beam silicon atoms. This mechanism is opposed to the crystallization mechanism in LPCVD polysilicon films, in which the crystallization process is driven by the thermal energy from substrate heating and occurs at >570 °C. (ii) E-beam-evaporated silicon atoms stick better to the SiO_2_ surface than to the Si surface; therefore, the initial silicon atoms deposited at the Si/SiO_2_ interface are likely to be less mobile on the silicon dioxide surface than the subsequently deposited silicon surface. As a result, the crystal formation induced by the kinetic energy from e-beam evaporation is impeded at the Si/SiO_2_ interface, creating a layer of amorphous silicon at and near the interface. Since the as-deposited amorphous silicon films display large residual tensile stress, films that are not fully crystallized with crystalline grains on top of the amorphous layer tend to bend downward when released. Phosphorus-doped UHVEE silicon films are mostly crystalline, with a minimal amorphous silicon layer at the interface due to the enhancement of crystallization as compared to boron and undoped films. Thus, these films exhibit significantly reduced and slightly negative stress gradients. When the amorphous silicon layer at the interface crystallizes, competitive grain growth occurs, which leads to the development of compressive stress at the silicon layer near the Si/SiO_2_ interface with randomly oriented grains. Conversely, the grains farther from the interface have columnar structures and relatively low compressive stresses. A higher compressive stress at the interface results in the film experiencing a small positive stress gradient.

Notably, the average residual stress of the UHVEE polysilicon changes gradually with respect to the deposition temperature due to early crystallization. This gradual change allows the film stress to be easily controlled in comparison to a LPCVD film, which exhibits drastic stress changes from 700 MPa to −500 MPa when the deposition temperature increases by 30 °C (from 600 °C to 630 °C).

Generally, fully crystalline UHVEE polysilicon films exhibit low average stresses and stress gradients at low thermal budgets. If a particular application allows a high thermal budget, the average stress and stress gradient in a fully crystalline UHVEE polysilicon film can be further minimized and even completely removed by annealing at high temperatures. This behavior of UHVEE polysilicon is similar to that of polysilicon films deposited by other techniques, such as LPCVD polysilicon.

The characteristics of the UHVEE silicon films may be influenced by the material on which they are deposited. The deposition attributes depend on the sticking coefficient of evaporated silicon on the material (whether the sticking coefficient is higher than silicon); the crystallization state of the material (amorphous or crystalline); and the surface roughness of the material (smooth or rough). The material used in this work is silicon dioxide, which is amorphous and smooth and has a higher sticking coefficient than silicon. Silicon nitride is also amorphous, can be deposited smoothly and has a similar sticking coefficient to silicon dioxide. Therefore, the qualities of the films deposited on smooth oxide and silicon nitride materials should be similar. To confirm this assumption, we deposit a UHVEE silicon film on smooth PECVD silicon nitride at a substrate deposition temperature of 500 °C and a deposition rate of 100 nm/min with phosphorus doping of 5 × 10^18^/cm^3^. The measured characteristics of the film, including the average residual stress, stress gradient, surface roughness, and film thickness, are listed in Table 2 as UHVEESi (SiN) technology. These values are close to those obtained for polysilicon evaporated on silicon dioxide.

**Table 2:** Comparison of UHVEE PolySi with other polysilicon technologies

Technologies	ARS (MPa)	SG (MPa/µm)	t (µm)	CT (°C)	DR (nm/min)	R (nm)	Electrically active
**Sputtering**	20–150^29^	>100^29^	< 5^29^	>700^29,30^	37^29^, 45^30^	1.8^30^, 5.9^29^	No
**PECVD SiGe**^a^	−200–250^36^	>6^36^	< 10^36^	400–600^36,38^	100–200^38^	117^38^	Yes
**LPCVD SiGe**^a^	−53–−228^12^	19–134^12^	< 2^12^	400–500^12^	5–13^12^	—	Yes
**LPCVD Si**	−450^25^–300^22^	>100^22^	< 1^25^	570–625^22,25^	2–30^22,25,37^	71^22^	Yes
**PECVD Si**	−450-50^37^	—	12^37^	>600	40–160^37^	—	No
**Epi-poly**	3^23^	2^23^	>10^23^	>1000^23^	>500^23^	3000^23^	Yes
**UHVEESi (SiN)**^a^	**86**	**4**	**4**	**500**	**100**	**6**	**Yes**
**UHVEESi**^a^	**−92-41**	**1.2-3**	**60**	**400—500**	**100–1000**	**<7**	**Yes**

*ARS* average residual stress, *SG* stress gradient, *t* thickness, *CT* crystallization temperature, *DR* deposition rate (DR), *R* roughness

^a^Fully crystallized low thermal budget technologies

Table 2 presents a comparison of the performance characteristics of major silicon deposition technologies to highlight the advantages and unique capabilities of the UHVEESi technology reported in this work. The PECVD SiGe, LPCVD SiGe and UHVEESi technologies are compatible with low thermal budget applications where full crystallization occurs at less than or equal to 500 °C. Epi-poly^23^ can only be achieved above 1000 °C and yields a very rough film. PECVD Si^37^ and sputtered Si^29,30^ require high-temperature annealing above 600 °C for full crystallization. Epi-poly, PECVD Si and sputtered Si are not suitable for enabling the formation of electrically active silicon films at low thermal budgets. The PECVD SiGe, LPCVD SiGe and UHVEESi technologies can be used to prepare electrically active silicon with a low thermal budget. By comparing these technologies, we find that the UHVEESi technology reported in this work has the advantages of enabling the deposition of low-stress, low-stress-gradient, thick, and smooth silicon films with low thermal budgets. We can deposit UHVEESi films with thicknesses reaching 60 µm. This deposition is not possible with either LPCVD or PECVD SiGe. LPCVD SiGe^12^ cannot be used to prepare silicon films with thicknesses greater than 2 µm. In addition, PECVD SiGe is not appropriate for silicon films thicker than 10 µm, and it yields rough silicon films.

## Materials and methods

### Ultra-high vacuum e-beam evaporation system

**Fig. 7:**
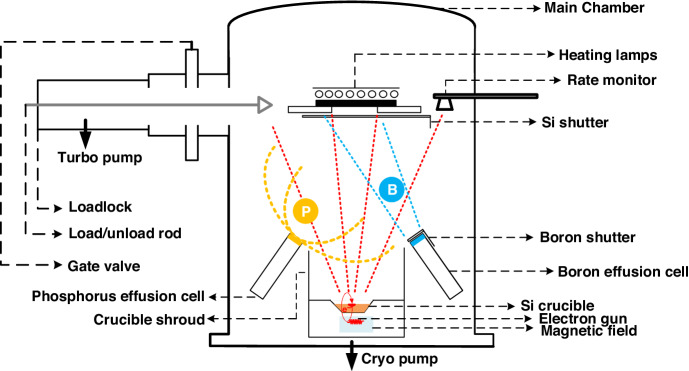
Ultrahigh-vacuum e-beam evaporation system used in this work

An in-house constructed ultrahigh-vacuum e-beam evaporation system is used to deposit the silicon films. The set-up of the system is schematically illustrated in Fig. 7. The system has multiple components. (i) To begin, the system features a vacuum pumping module to achieve a base pressure in the order of 10^−9^ Torr in the main deposition chamber. During evaporation, the pressure in the chamber increases to the order of 10^−8^) Torr depending on the deposition rate and substrate temperature. The vacuum pumping module consists of a CTI-10 high-vacuum cryopump with a helium compressor with auto pumping, venting and regeneration functionalities, a dry vacuum pump (not shown), and a main chamber bakeout system that includes a halogen lamp and isolation shielding. The vacuum system has all the necessary vacuum gauges, valves, controllers, sensors, and embedded electronics. (ii) A single e-beam gun source (10 kV, 1 A) equipped with a single large crucible pocket size of 100 cc is used to allow many evaporations of thick films before refill. An electronic grade silicon ingot (resistivity ρ > 2700 Ω.cm) in the form of a pellet is used in a crucible as a silicon source for evaporation. To deposit the films at 50 nm/min, 100 nm/min and 400 nm/min, the voltage is held constant at 9.2 kV, and the currents are set to 130 mA, 160 mA and 260 mA, respectively. The Si deposition rate is measured by a rate monitor. (iii) The load lock module consists of a load lock chamber, turbo pump system and manual transfer arm (load/unload rod). The load lock system is critically important for maintaining UHV operation in the main chamber and reducing the pumping period. The load lock chamber allows a substrate size of 4” to be manually loaded and transferred to the main chamber after the load lock is pumped down to 5e^−7^ Torr. (iv) A quartz heating lamp is used to control the substrate deposition temperature to a maximum sample size of 4”. The heater allows a substrate temperature reaching 650 °C to be set and controlled. (v) A Planar 4” substrate holder with substrate rotation (0–30 rpm) and a temperature gauge to control the substrate temperature is used. (vi) A Si shutter can be opened and closed to control deposition on the sample mounted on the substrate holder. (vii) Deposition on the substrate mounted on the substrate holder is controlled by a Si shutter, which can be opened or closed. (viii) An in situ doping module is used that consists of a CreaTec TUBO source for boron doping from boron material and a DECO source for phosphorus doping from the decomposition of the GaP compound. The effusion cells have a 10-cc charge capacity, and their operating temperatures are calibrated and controlled to obtain the desired doping levels. The effusion cells should be calibrated regularly to precisely control the dopant flux. Secondary ion mass spectrometry (SIMS) is a very helpful tool for this purpose.

### Polysilicon film evaporation

Silicon substrates are first thermally oxidized to form a thermal oxide with a thickness of 1 μm. Then, UHVEE silicon films are evaporated onto the substrates at various temperatures (200 °C–625 °C) at a deposition rate of 100 nm/min. Furthermore, to study the effects of the deposition rate on the silicon films, the deposition rate is varied to values of 50 nm/min, 100 nm/min, 200 nm/min, and 400 nm/min at 500 °C. In addition, the effects of introducing dopants into the silicon film are investigated. Boron-doped films with 5 × 10^18^/cm^3^ boron concentrations and phosphorus-doped films with 5 × 10^18^/cm^3^ and 2 × 10^19^/cm^3^ phosphorus concentrations are deposited and studied.

The as-deposited UHVEE silicon films are characterized for their fraction of crystallization relative to the substrate deposition temperature, predominant silicon crystal orientation, microstructural texture, surface morphology, residual stress, and Young’s modulus. The fraction of crystallization is measured using Raman spectroscopy. The crystallographic orientations are characterized using X-ray diffraction (XRD). Focus-ion beam (FIB) technology is used to prepare a thin sample for cross-sectional transmission electron microscopy (X-TEM) to analyze the microstructural texture. Atomic force microscopy (AFM) is used to measure the surface morphology. The stress is calculated by measuring the curvature of the silicon substrates before and after UHVEE silicon deposition. Cantilever beams are patterned, etched with deep reactive ion etching (DRIE) and released. The deflection profiles and resonance frequencies of the released cantilevers are measured with a Polytec MSA500 to evaluate the stress gradient and Young’s modulus parameters of the films.

### Comb-drive fabrication

**Fig. 8:**
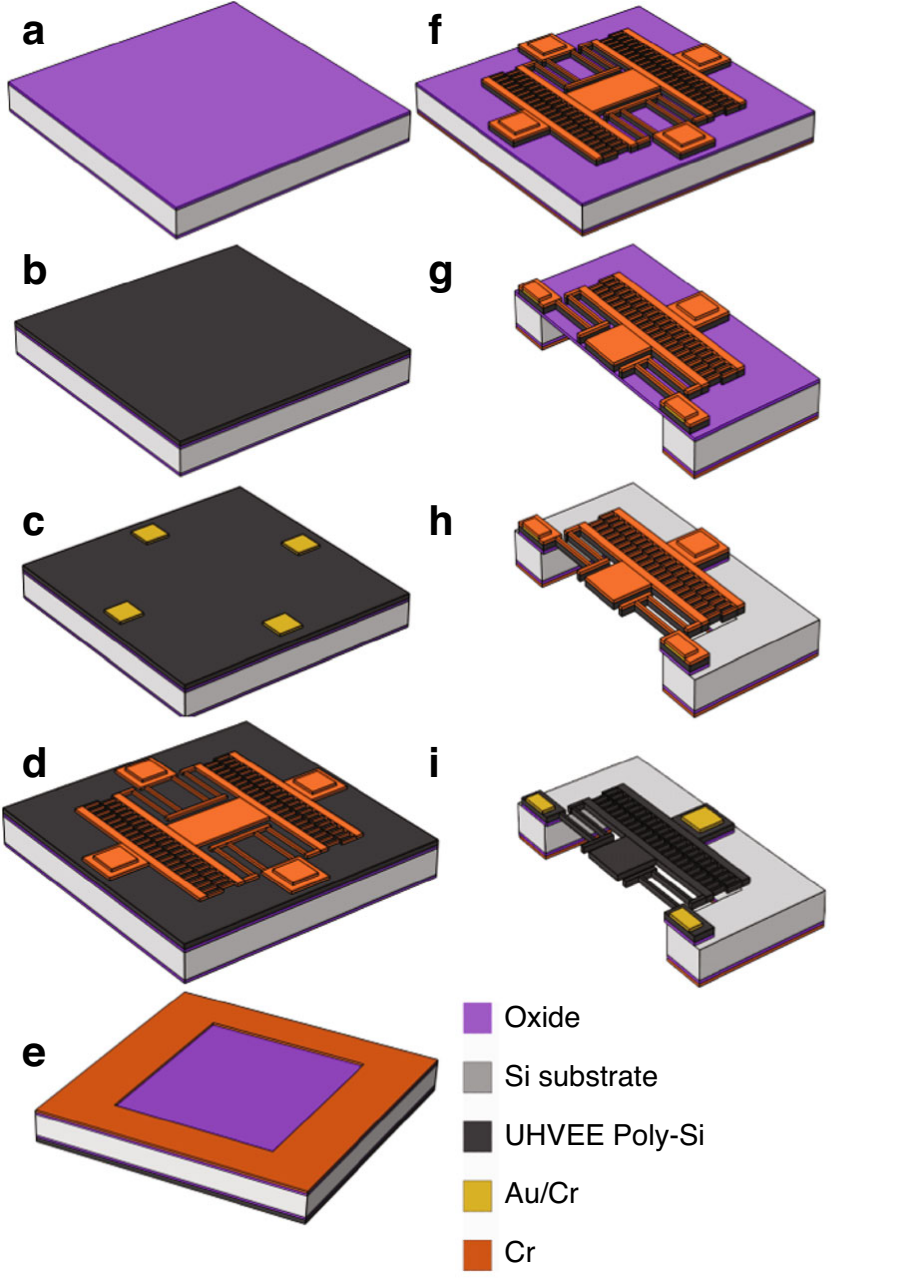
Comb-drive fabrication. **a** Grow oxide; **b** deposit thick UHVEEPolySi; **c** electrical pads patterned; **d** pattern the comb-drive; **e** backside pattern; **f** DRIE of UHVEEPolySi; **g** STS ICP oxide and DRIE from backside; **h** Remove Cr using O_2_ plasma; **i** HF vapor etch

On top of the oxidized silicon wafers (Fig. 8a), 20-μm-thick in situ phosphorus-doped polysilicon is deposited at a rate of 400 nm/min with 5 × 10^18^/cm^3^ phosphorus doping at a substrate deposition temperature of 450 °C (Fig. 8b).

A dual layer of Au/Cr with a thickness of 46 nm/4 nm is e-beam evaporated and lifted-off in NMP solution to form the metal pads (Fig. 8c). Following similar processing steps, a layer of 30-nm-thick Cr is patterned to define the comb-drive pattern and protect the Au/Cr electrodes from reactive ion etching during the etching process (Fig. 8d). The front side of the sample is then protected by a thin layer of positive photoresist. The wafer is flipped, and another layer of positive photoresist is spun on the backside. The photoresist is patterned, followed by chromium evaporation and lift-off, defining the backside etching windows (Fig. 8e). The frontside protective photoresist is removed when the chromium layer is removed from the NMP solution. The front side is etched to form high-aspect-ratio comb-drive fingers (Fig. 8f). The sample is flipped over, and deep reactive ions are etched to release thermal oxide diaphragms on the substrate featuring the comb-drive structure (Fig. 8f). The comb structure is then released by HF vapor etching, which removes the oxide (Fig. 8h). Finally, oxygen ash with a biased platen is applied to remove the chromium mask, expose the gold electrodes and clear the sidewall polymer residuals (Fig. 8i).

## References

[1] Bryzek J (1996). Impact of MEMS technology on society. Sens. Actuators A: Phys..

[2] Fedder GK, Howe RT, Liu T-JK (2008). Technologies for Co-fabricating MEMS and Electronics. Proc. IEEE.

[3] Sedky S, Witvrouw A, Bender H, Baert K (2001). Experimental determination of the maximum post-process annealing temperature for standard CMOS wafers. IEEE Trans. Electron Devices.

[4] Takeuchi H, Wung A, Xin S, Howe RT, Tsu-Jae K (2005). Thermal budget limits of quarter-micrometer foundry CMOS for post-processing MEMS devices. IEEE Trans. Electron Devices.

[5] Brunet, L. et al. Breakthroughs in 3D sequential technology. in 2018 IEEE international Electron Devices Meeting (IEDM) 7.2.1-7.2.4 2018.

[6] French PJ (2002). Polysilicon: a versatile material for microsystems. Sens. Actuators A: Phys..

[7] Brazzle JD, Taylor WP, Ganesh B, Price JJ, Bernstein JJ (2003). A hysteresis-free platinum alloy flexure material for improved performance and reliability of MEMS devices. TRANSDUCERS ‘03. 12th Int. Conf. Solid-State Sens., Actuators Microsyst. Dig. Tech. Pap. (Cat. No. 03TH8664)..

[8] Yamane, D. et al. An arrayed MEMS accelerometer with a wide range of detection. *2013 Transducers & Eurosensors XXVII: The 17th International Conference on Solid-State Sensors, Actuators and Microsystems (TRANSDUCERS & EUROSENSORS XXVII)*. 22-25 (Barcelona, Spain, 2013).

[9] Huang, W. Ren, Z. & Nguyen, C. T. Nickel Vibrating Micromechanical Disk Resonator with Solid Dielectric Capacitive-Transducer Gap. *IEEE International Frequency Control Symposium and Exposition*. 839–847 (Miami, FL, USA, 2006)

[10] Low, C. W. Almeida, S. F. Quevy, E. P. Howe, E. T. Poly SiGe Surface Micromachining in 3D and IC integration of MEMS. Eshashi, M. Editor. *Wiley. VCH*. (2021)

[11] Witvrouw, A. et al. Processing of MEMS gyroscopes on top of CMOS ICs in *Proc. IEEE Int*. *Solid-State Circuits Conf*. 88–89 (2005).

[12] Low CW, Liu TK, Howe RT (2007). Characterization of Polycrystalline Silicon-Germanium film deposition for modularly integrated MEMS applications. J. Microelectromechanical Syst..

[13] Becker C (2009). Microstructure and photovoltaic performance of polycrystalline silicon thin films on temperature-stable ZnO:Al layers. J. Appl. Phys..

[14] Sontheimer T, Scherf S, Klimm C, Becker C, Rech B (2011). Characterization and control of crystal nucleation in amorphous electron beam evaporated silicon for thin film solar cells. J. Appl. Phys..

[15] Michael, A. Kwok, C. Y. Wang, P. Kazuo, O. & Varlamov, S. Investigation of E-Beam Evaporated Silicon Film Properties for MEMS Applications. 9, (2015).

[16] Houben L, Luysberg M, Hapke P, Carius R, Finger F, Wagner H (1998). Structural properties of microcrystalline silicon in the transition from highly crystalline to amorphous growth. Philos. Mag. A.

[17] Kamins TI (1980). Structure and Properties of LPCVD Silicon Films. J. Electrochem. Soc..

[18] Kinsbron E, Sternheim M, Knoell R (1983). Crystallization of amorphous silicon films during low pressure chemical vapor deposition. Appl. Phys. Lett..

[19] Wada Y, Nishimatsu S (1978). Grain growth mechanism of heavily phosphorus‐implanted polycrystalline silicon. J. Electrochem. Soc..

[20] Veprek S, Sarott FA, Iqbal Z (1987). Effect of grain boundaries on the Raman spectra, optical absorption, and elastic light scattering in nanometer-sized crystalline silicon. Phys. Rev. B.

[21] Jie Y, Kahn H, An-Qiang H, Phillips SM, Heuer AH (2000). A new technique for producing large-area as-deposited zero-stress LPCVD polysilicon films: the MultiPoly process. J. Microelectromechanical Syst..

[22] French PJ, Evans AGR (1989). Piezoresistance in polysilicon and its applications to strain gauges. Solid-State Electron..

[23] Wu G, Xu J, Ng EJ, Chen W (2020). MEMS resonators for frequency reference and timing applications. J. Microelectromechanical Syst..

[24] Sosale G, Almecija D, Das K, Vengallatore S (2012). Mechanical spectroscopy of nanocrystalline aluminium films: effects of frequency and grain size on internal friction. Nanotechnology.

[25] Maier-Schneider D, Köprülülü A, Holm SB, Obermeier E (1996). Elastic properties and microstructure of LPCVD polysilicon films. J. Micromech. Microeng..

[26] S. D. Senturia, S. D. Microsystem design. *Springer Science & Business Media*, 2007.

[27] Carlen ET, Khee-Hang H, Bakshi S, Pareek A, Mastrangelo CH (2005). High-aspect ratio vertical comb-drive actuator with small self-aligned finger gaps. J. Microelectromechanical Syst..

[28] Seok S, Lee B, Kim J, Kim H, Chun K (2005). A new compensation method for the footing effect in MEMS fabrication. J. Micromech. Microeng..

[29] Honer KA, Kovacs GTA (2001). Integration of sputtered silicon microstructures with pre-fabricated CMOS circuitry. Sens. Actuators A: Phys..

[30] Chandra S, Pal P (2005). RF sputtered silicon for MEMS. J. Micromech. Microeng..

[31] Hatalis MK, Greve DW (1988). Large grain polycrystalline silicon by low‐temperature annealing of low‐pressure chemical vapor deposited amorphous silicon films. J. Appl. Phys..

[32] Thompson CV (1987). Grain growth in polycrystalline silicon films. MRS Proc..

[33] Bisaro R (1985). Solid-phase crystallization kinetics in doped a-Si chemical-vapor-deposition films. Phys. Rev. B.

[34] Krulevitch P, Johnson GC, Howe RT (1991). Stress and microstructure in Lpcvd polycrystalline silicon films: experimental results and closed form modeling of stresses. MRS Proc..

[35] Guckel H, Sniegowski JJ, Christenson TR, Raissi F (1990). The application of fine-grained, tensile polysilicon to mechanicaly resonant transducers. Sens. Actuators A: Phys..

[36] Sedky S, Witvrouw A, Baert K (2002). Poly SiGe a promising material for MEMS monolithic integration with the driving electronics. Sens. Actuators A: Phys..

[37] Iliescu C, Chen B (2004). Thick and Low-Stress PECVD amorphous silicon for MEMS applications. J. Micromech. Microeng..

[38] Rusu C (2003). New Low-Stress PECVD PolySiGe Layers for MEMS. J. Microelectromechanical Syst..

